# Elevated Serum MicroRNA Levels Associate with Absence of High-Grade Prostate Cancer in a Retrospective Cohort

**DOI:** 10.1371/journal.pone.0124245

**Published:** 2015-04-14

**Authors:** Brittany L. Mihelich, Joseph C. Maranville, Rosalie Nolley, Donna M. Peehl, Larisa Nonn

**Affiliations:** 1 Department of Pathology, University of Illinois at Chicago, Chicago, IL, 60612, United States of America; 2 Clinical Pharmacology and Pharmacogenomics, University of Chicago, Chicago, IL, 60637, United States of America; 3 Department of Urology, Stanford University, Palo Alto, CA, 94305, United States of America; Roswell Park Cancer Institute, UNITED STATES

## Abstract

To reduce treatment of indolent prostate cancer (PCa), biomarkers are needed to improve identification of patients with a low-risk of having aggressive disease. Over-treatment of these patients occurs because of uncertainty in the aggressiveness of the entire tumor based on the biopsies, which do not accurately sample multifocal tumors. Circulating microRNAs (miRNAs) are stable serum markers and differential miRNA levels occur in men with PCa. The goal of this study was to identify circulating miRNAs that were associated with aggressive or indolent PCa. We measured circulating miRNAs in 150 patients prior to surgery and compared the miRNA levels to the pathology of the entire radical prostatectomy specimen. For this study we used an exceptionally well-characterized cohort of patients who had benign prostatic hyperplasia (BPH), low-grade or high-grade PCa. Low-grade was defined as patients with 100% Gleason grade 3 tumor as determined by step-wise sectioning. High-grade PCa patients had 30-90% Gleason grade 4+5 in the tumor. BPH patients had at least two biopsies negative for PCa. Twenty one miRNAs were selected for analysis. The miRNAs were quantified by RT-qPCR and analyzed by logistic regression. High levels of 14 miRNAs were exclusively present in the serum from patients with low-grade PCa or BPH, compared to men with high-grade PCa who had consistently low levels. The expression levels of the 14 miRNAs were combined into a “miR Score” which had a negative predictive value (NPV) of 0.939 to predict absence of high-grade PCa among PCa and BPH patients. Biochemical recurrence (BCR) was known for the PCa patients and a combined “miR Risk Score” accurately classified a subset of patients with low risk of BCR (NPV 0.941). In summary, measurement of serum miRNAs may have pre-surgical utility in combination with clinical risk calculators to identify patients with low risk of harboring aggressive PCa.

## Introduction

Although prostate cancer (PCa) remains the second leading cause of cancer death, only ~10% of the 233,000 expected men who will be diagnosed with PCa in 2014 will have potentially lethal disease [1]. PSA (prostate specific antigen) screening has increased PCa diagnosis and decreased PCa mortality overall, but low levels of PSA are not predictive of tumor aggressiveness and there is controversy over using PSA to detect indolent PCa [2, 3]. Patients with indolent PCa and a low-risk of having aggressive disease may be monitored safely by active surveillance [4, 5], but there can be uncertainty with this determination. Considering the significant morbidities associated with PCa treatment (impotence, incontinence, pain, infertility) improved risk stratification is needed to identify these low-risk patients and reduce treatment of indolent PCa.

The uncertainty in PCa prognosis is due the fact the prostate biopsies do not adequately sample the prostate tumor, thus the trend is to treat most men with PCa to avoid not treating a man who had aggressive disease that was missed by the biopsy. This is a valid concern as upgrading after prostatectomy occurs in 30–50% of men with Gleason sum 3+3 = 6 biopsies [6]. Gleason grade is determined by a pathologist and is reflective the degree of differentiation of PCa and is associated with its aggressiveness. Gleason grades range from 1 to 5, of which 5 is the highest, most aggressive, grade. Pathologists often report the Gleason sum (or score) of the most prevalent grade and the second most prevalent grade. The risk of cancer recurrence after radical prostatectomy increases proportionally with the percent of Gleason grades 4 or 5 in the tumor, whereas patients with confirmed 100% Gleason grade 3 (i.e., Gleason sum 3+3 = 6) cancer rarely recur [7] and can be safely monitored by active surveillance [4, 5]. Therefore, Gleason sum 3+3 = 6 patients who may not benefit from surgical or radiation treatment [4, 5], and may suffer decreased quality of life because PCa treatment.

There are several pre-surgical risk assessment calculators [8, 9] available that incorporate clinical variables to estimate risk of biochemical recurrence (BCR) post-radical prostatectomy. While these calculators are useful for clinical management of PCa patients with the highest risk of BCR, patients with low or intermediate risk (i.e., Gleason sum, 3+3, 3+4 or 4+3) remain a clinical challenge.

MicroRNAs (miRNAs) are attractive biomarkers of disease as they are both easily detected and stable in serum [10, 11]. MiRNAs are small, non-coding RNAs that post-transcriptionally regulate gene expression via binding to the 3’ untranslated region of target messenger RNAs [12]. In addition to this intracellular function, cell-free serum miRNAs have been found to be differentially present in the sera of PCa patients compared to healthy controls in several profiling studies [10, 13, 14] and in PCa patients with and without metastases [11]. However, there is little overlap in the miRNAs identified between studies, possibly because of small numbers of subjects or differences in study design and methodologies [reviewed in [15]]. Circulating miRNA biomarkers of PCa diagnosis and metastasis are becoming well established [16–22], but not yet used in the clinic.

Given the clinical challenges in PCa prognosis, there is yet to be identified a prognostic serum miRNA biomarker for clinically-relevant aggressive PCa, which would be valuable in reducing the overtreatment of indolent PCa. Therefore, herein we describe analysis of miRNAs in pre-surgical serum to discover a miRNA signature that associates with PCa Gleason grade. To assess the diagnostic and prognostic value of pre-surgical serum miRNAs, we profiled the levels of 21 miRNAs in 50 men with BPH and 100 men with PCa, 50 of whom had 100% Gleason grade 3 tumors and no BCR and 50 men with tumors with 30–90% Gleason grade 4 or 5 and variable BCR. None of the patients had been treated for disease at the time of serum collection. The 21 miRNAs were selected from the current literature at the time as being detectable in the serum of PCa patients. Using this dichotomous cohort, we sought to identify serum miRNAs capable of discriminating Gleason grade and to predict BCR.

## Materials and Methods

### Patients and Specimens

Sera were obtained with informed consent under an IRB-approved protocol at Stanford University 1998–2004 and stored at -80° C. Sera from 100 men with PCa were obtained during the pre-operative consultation several days prior to surgery. Patients received no treatment prior to surgery and serum collection. Patient characteristics and clinicopathologic variables were obtained from an existing database. Patient race was not recorded, but the patient population at Stanford during that time period was ~97% Caucasian. Sera from 50 men with BPH were obtained during office visits for clinical evaluation during this same time period and absence of PCa was confirmed by at least two sets of ultrasound-guided needle biopsies.

### RNA Extraction, Reverse Transcription and qPCR

Serum samples were coded and blinded and RNA was extracted from 250 μl of serum with miRNeasy (Qiagen, Valencia, CA). RNA (4 μl) was reverse transcribed using the Universal cDNA Synthesis Kit (Exiqon Inc., Denmark). Exogenous oligonucleotides were spiked into the samples prior to each step to control for variation in RNA extraction (cel-miR-39), cDNA synthesis (Sp6), and inter-plate calibration (Sp3). qPCR was run on a ViiA 7 Real-Time PCR System (Applied Biosystems, Foster City, CA) using Exiqon SYBR green and custom Pick&Mix miRNA PCR plates containing primers for 21 miRNAs of interest and the controls Sp3, Sp6, and cel-miR-39 (Exiqon, Inc). Wells with Ct>37 or poor melting curves were excluded from analysis.

### Statistical Analysis

#### Normalization of PCR data

Ct values were normalized to the Sp3 spike-in for plate-to-plate PCR variability. To control for cDNA syntheses and RNA extraction efficiencies, Ct values were further normalized to Sp6 and cel-miR-39, respectively. Finally, samples were normalized to the total RNA input. There were no statistical differences between the analysis groups in the exogenous controls or RNA inputs (**S1A Fig**). Exiqon-recommended endogenous normalizers let-7a and miR-103a were significantly different between groups and therefore not utilized (**S1B Fig**). Relative quantities (RQ) were calculated by a modified ΔCt method [23] with normalization to RNA input.

#### Differences between groups

RQs were log2 transformed to approximate a normal distribution. Analyses were performed in R statistical package. Wilcoxon rank sum test or Kruskal–Wallis one-way analysis of variance with Dunn's multiple comparisons test was used to assess differences between groups.

#### miR Scores for Gleason grade

All miRNAs significantly different between the groups were incorporated into miR Scores. Log odds ratios representing the change in natural log odds of Gleason grade 3, grade 4+5, or BPH status per 2-fold increase in miRNA expression level were calculated by logistic regression. These estimated log odds ratios were then multiplied by the log2 RQ levels for each miRNA in each patient. This resulted in the two miR Scores; miR Score1, which was optimized to separate low-grade from high-grade PCa patients, and miR Score2, which was optimized to separate BPH and low-grade PCa from high-grade PCa. The equations for the miR Scores, in which the miRNA name represents the log2 RQ value: miR Score1 = (0.269 x let-7a) + (0.257 x miR-103) + (0.254 x miR-451) + (0.255 x miR-24) + (0.252 x miR-26b) + (0.255 x miR-30c) + (0.221 x miR-93) + (0.253 x miR-106a) + (0.274 x miR-223) + (0.188 x miR-874) + (0.200 x miR-146a) + (0.118 x miR-100) + (0.276 x miR-107) + (0.204 x miR-130b). miR Score2 = (0.289 x level Let7a) + (0.286 x level miR-103) + (0.308 x level miR-451) + (0.267 x level miR-24) + (0.256 x level miR-26b) + (0.282 x level miR-30c) + (0.231 x level miR-93) + (0.263 x level miR-106a) + (0.293 x level miR-223) + (0.161 x level miR-874) + (0.227 x level miR-146a) + (0.165 x level miR-125b) + (0.116 x level miR-100) + (0.295 x level miR-107) + (0.183 x level miR-130b).

The positive predictive value (PPV) and negative predictive value (NPV) across all possible thresholds were estimated (using each ranked miR Score as a threshold). The miR Score thresholds were cross-validated by two methods. First, leave-one-outcross-validation (LOOCV) was run. Briefly, one patient at a time was removed from the data set, and then log odds ratios were re-estimated for each miRNA in the remaining patients. The ability of the new miR Score to properly classify the removed patient was then assessed. Second, two-fold cross-validation (2-fold CV) approach was used in which each patient was randomly assigned to a training or validation set of equal size (n = 50). Log odds ratios were estimated in the training set and the PPV and NPV of miR Scores were assessed in the validation set based on these estimates. This random sampling was repeated and PPV and NPV were estimated in 10,000 independent iterations.

To assess the ability of the miR Score to distinguish Gleason grade 3 or BPH patients from those with grade 4 + 5 cancer across the range of scores, a Receiver-Operator Characteristic curve was generated using the software ‘pROC’[24].

#### miR Risk Score for BCR

A similar approach as described above was used to identify a miR Risk Score for BCR. Briefly, the log odds ratio of risk of BCR was estimated for each significantly associated miRNA and these values were used to calculate a miR Risk Score using the equation: miR Risk Score = (0.223 x miR-451) + (0.225 x miR-106a) + (0.217 x miR-223) + (0.215 x miR-107) + (0.147 x miR-130b) + (0.217 x of let-7a) + (0.196 x of miR-26b). A threshold of the PPV and NPV for BCR-free survival was determined and cross-validated as described above.

#### Kaplan-Meier analysis

BCR-free survival times were examined for the PCa patients above and those below the threshold miR Risk Score of 3.26. Kaplan-Meier curves were plotted using the packages ‘KMsurv’ and ‘survival’ for all patients and those with CAPRA-intermediate status. Patients above and below the mean were compared using log rank tests.

### Cell cultures

The isogenic human prostate epithelial cell lines RWPE-1 and RWPE-2 were obtained from ATCC and maintained in KSFM with BPE and EGF (Gibco). Primary prostatic epithelial cells were derived from benign (PrE) and 100% Gleason 4 tumor (PrE-Ca) areas of a radical prostatectomy specimen at UIC via IRB-approved protocol. PrE and PrE-Ca cells were maintained in PrEGM (Lonza) as previously described [25, 26]. Cells were collected at 75% cell density 24 hours after feeding. RNA isolation and RT-qPCR analysis was as described above. MiRNAs were normalized to RNU 44 and 48.

## Results

### Circulating miRNA levels differentiate BPH and low-grade PCa from high-grade PCa

RNA was isolated from sera from 50 men with 100% Gleason grade 3 PCa tumors (low-grade), 50 with tumors containing 30–90% Gleason grade 4 and/or 5 PCa (high-grade), and 50 with BPH (**Table 1**). We measured the levels of 21 miRNAs, selected from our own work and literature reports, current at the time of our experiments, of differential levels in sera or cancer tissues of PCa patients compared to non-cancer controls [10, 11, 25–28] (**Table 2**). miRs-96, -141, 182, 183 were not detectable in >50% of the patients, had average Ct>35 and there was no pattern by disease (**S1 Dataset**). miR-1274a, detectable in all sera, is no longer considered a miRNA, but is a fragment of Lys tRNA [29].

**Table 1:** Patient Demographics.

* *		BPH	Prostate Carcinoma PatientsPost-RP Pathology		P Value
			100% Gleason grade 3	30–90% Gleason grade 4+5	
**Total**		50	50	50	
**Age**	Range	56–80	46–75	46–78	
	Mean	66^a^	62^a^	64	0.0012
**PSA**	Range	1.33–23.31	1.2–35.8	2.62–490	
	Mean	9.9	7.9	24.7	0.007
**Stage, Clinical** ^**b**^	T1C	N/A	33	25	
	T2A	N/A	11	9	
	T2B	N/A	5	12	
	T2C	N/A	1	4	0.117
**Gleason sum biopsy** ^**c**^	Range	N/A	6–7* (n = 30)	6–9 (n = 39)	
	Mean	N/A	6.04	7.1	
	Gleason 7		(3+4* n = 1)	(3+4 n = 10)(4+3 n = 9)	8.66x10^-13^
**Biochemical Recurrence** ^**d**^	No	N/A	50	23	
	Yes	N/A	0	27	1.13x10^-10^

^a^p = 0.0012, two-tailed t-test.

^b^Gleason 3 and Gleason 4+5 groups p = 0.117 by G-Test of Independence.

^c^Gleason 3 and Gleason 4+5 groups p = 8.7x10^-13, two-tailed t-test.

^d^Gleason 3 and Gleason 4+5 groups, p = 1.1x10^-10 Fisher Exact Test. BPH, Benign Prostatic Hyperplasia.

*Gleason 4 was detected in one patient on biopsy, but not observed in final surgical RP specimen.

**Table 2:** miRNAs measured in the serum and delectability in this cohort.

microRNA	Findings from this study		Original Publication
	Gleason 3 vs Gleason 4+5	Biochemical Recurrence	
miR-1274a	No longer considered miR		Moltzahn et al. 2010 (10)
miR-141	Detectable in <50% of patients		Bryant et al. 2012 (11)
mir-182	Detectable in <50% of patients		Mihelich et al. 2011 (20)
miR-183	Detectable in <50% of patients		Mihelich et al. 2011 (20)
miR-96	Detectable in <50% of patients		Mihelich et al. 2011 (20)
Let-7a	**p<0.05**	**p<0.05**	Henegan et al. 2010 (28)
miR-103	**p<0.05**	**p<0.05**	Kan et al. 2012 (27)
miR-107	**p<0.05**	**p<0.05**	Bryant et al. 2012 (10)
miR130b	**p<0.05**	**p<0.05**	Bryant et al. 2012 (10)
miR-106a	**p<0.05**	**p<0.05**	Moltzahn et al. 2010 (10)
miR-26b	**p<0.05**	**p<0.05**	Moltzahn et al. 2010 (10)
miR-451	**p<0.05**	**p<0.05**	Moltzahn et al. 2010 (10)
miR-223	**p<0.05**	**p<0.05**	Moltzahn et al. 2010 (10)
miR-93	**p<0.05**	NS	Moltzahn et al. 2010 (10)
miR-24	**p<0.05**	NS	Moltzahn et al. 2010 (10)
miR-30c	**p<0.05**	NS	Moltzahn et al. 2010 (10)
miR-874	**p<0.05**	NS	Moltzahn et al. 2010 (10)
miR-100	**p<0.05**	NS	Giangreco et al. 2013 (25)
miR-146a	**p<0.05**	NS	Giangreco et al. 2013 (25)
miR-125b	NS	NS	Giangreco et al. 2013 (25)
miR-1207-5p	NS	NS	Moltzahn et al. 2010 (10)

**NS =** Not significant p>0.05

**Fig 1:**
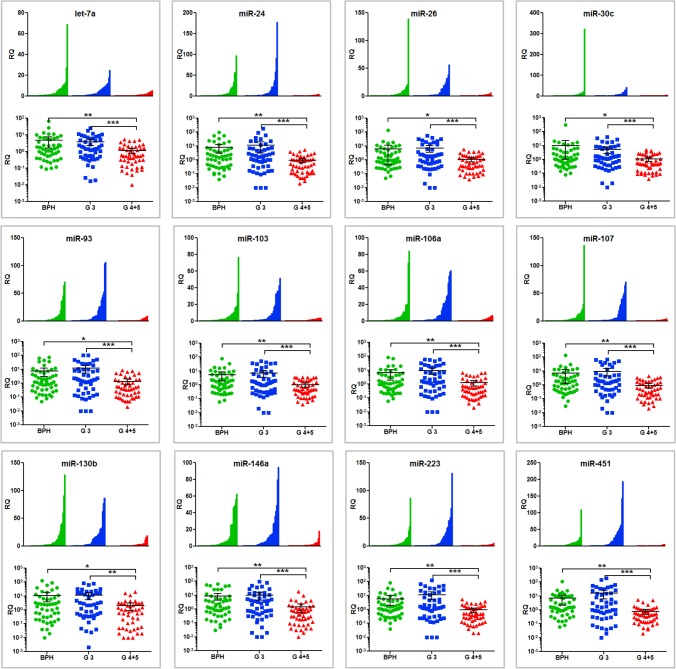
Circulating miRNAs levels in patients with BPH, low-grade PCa and high-grade PCa. Waterfall plots (linear y-axis) and scatter plots (log10 y-axis) of RQ values for all patients. In the waterfall plots each patient is shown as a vertical line on the x-axis and sorted by RQ within each group, for the top 12 miRNAs in the miR Score; let-7a, miR-24, 26a, 30c, 93, 103, 106a, 107, 130b, 146a, 223, and 451 and by disease. For the scatter plots the y-axis is shown log10 to improve visualization of low range RQs. BPH = benign prostate hyperplasia (N = 50). Gleason 3 = 100% of the tumor was Gleason grade 3 (N = 50). Gleason 4+5 = 30–90% of the tumor was Gleason 4 and/or 5 (N = 50).*p<0.05, ** p<0.01, *** p<0.0001 by Kruskal–Wallis one-way analysis of variance with Dunn's multiple comparisons test.

The remaining 16 miRNAs were detectable in our sera (**Table 2, S1 Dataset**) and 14 were detected at uniformly low levels in the high-grade PCa group, but were present at significantly higher and more heterogeneous levels in patients with low-grade PCa or BPH (12 top miRNAs are shown **Fig 1**). RNA recovery and spike-in controls did not vary between the groups (**S1A Fig**). None of the miRNAs were associated with gross serum hemolysis (**S2A and S2B Fig**). Ct values for all samples can be accessed in **S1 Dataset**.

**Fig 2:**
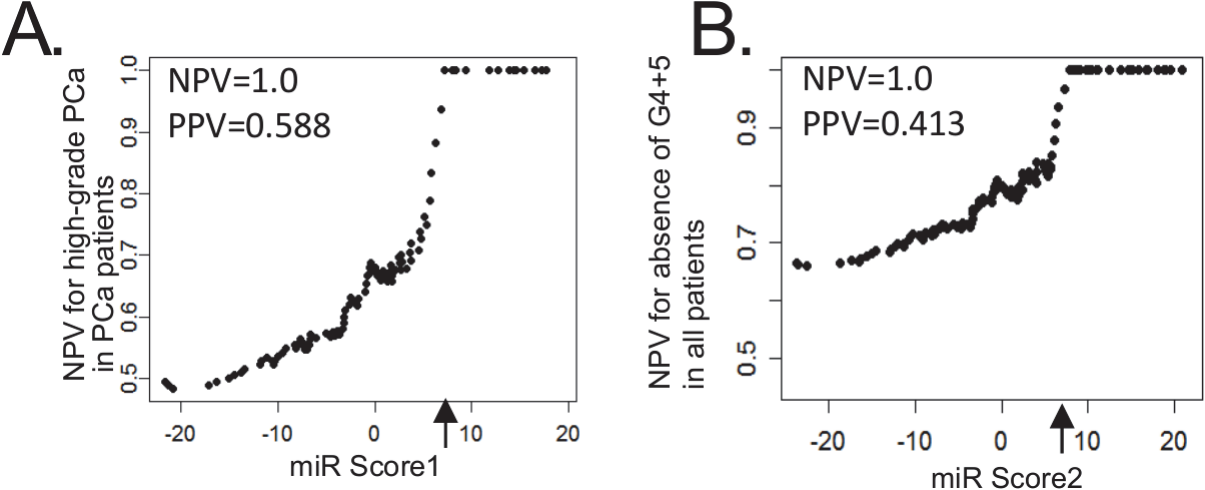
Circulating miRNAs predict absence of high-grade disease. **A,** negative predictive value (NPV, y-axis) for absence of Gleason 4 +5 in the PCa patients only (N = 100) across the range of miR Score1 (x-axis) reaching NPV = 1 at the miR Score1 threshold of 7.19 (arrow). **B**, NPV reaches 1 for absence of Gleason 4+5 in entire cohort (BPH, low-grade PCa, high-grade PCa) at the miR Score2 threshold of 7.85 (arrow). The equations for the miR Scores, in which the miRNA name represents the log2 RQ value: miR Score1 = (0.269 x let-7a) + (0.257 x miR-103) + (0.254 x miR-451) + (0.255 x miR-24) + (0.252 x miR-26b) + (0.255 x miR-30c) + (0.221 x miR-93) + (0.253 x miR-106a) + (0.274 x miR-223) + (0.188 x miR-874) + (0.200 x miR-146a) + (0.118 x miR-100) + (0.276 x miR-107) + (0.204 x miR-130b). miR Score2 = (0.289 x level Let7a) + (0.286 x level miR-103) + (0.308 x level miR-451) + (0.267 x level miR-24) + (0.256 x level miR-26b) + (0.282 x level miR-30c) + (0.231 x level miR-93) + (0.263 x level miR-106a) + (0.293 x level miR-223) + (0.161 x level miR-874) + (0.227 x level miR-146a) + (0.165 x level miR-125b) + (0.116 x level miR-100) + (0.295 x level miR-107) + (0.183 x level miR-130b).

The prognostic and diagnostic value of these fourteen miRNAs was examined by calculating a “miR Score” that included the RQ and odds-ratio for each of the miRNAs present at significantly different levels between the groups. For prognosis, we used “miR Score1” to distinguish low-grade from the high-grade PCa patients. The threshold of 7.19 had a NPV of 1 and PPV of 0.588, thus highly predictive of low-grade PCa (**Fig 2A**). LOOCV and 2-fold CV showed similar results with NPVs of 0.938 and 1±0.15, respectively, and PPVs of 0.583 and 0.564±0.06, respectively, for low-grade PCa. The AUC for low-grade PCa was 0.69 across the range of miR risk scores (**S3A Fig**).

To test the miRNAs as markers of diagnosis, the significantly different miRNAs were combined into “miR Score2” to predict the absence of high-grade PCa in the BPH and low-grade PCa patients. The miR Score2 included the same miRNAs as miR Score1 plus miR-125b. A threshold of 7.85 for miR Score2 was highly predictive of BPH or low-grade PCa, with a NPV of 1 and PPV of 0.413 (**Fig 2B**). Cross validation of the miR Score by LOOCV (NPV = 0.938 and PPV = 0.366) and 2-fold CV (1±0.14) and NPV = 0.564±0.04) showed similar results for absence of high-grade PCa. The AUC for absence of high-grade disease was 0.69 across the range of miR risk scores (**S3B Fig**).

### miRNAs levels predict disease-free survival

**Fig 3:**
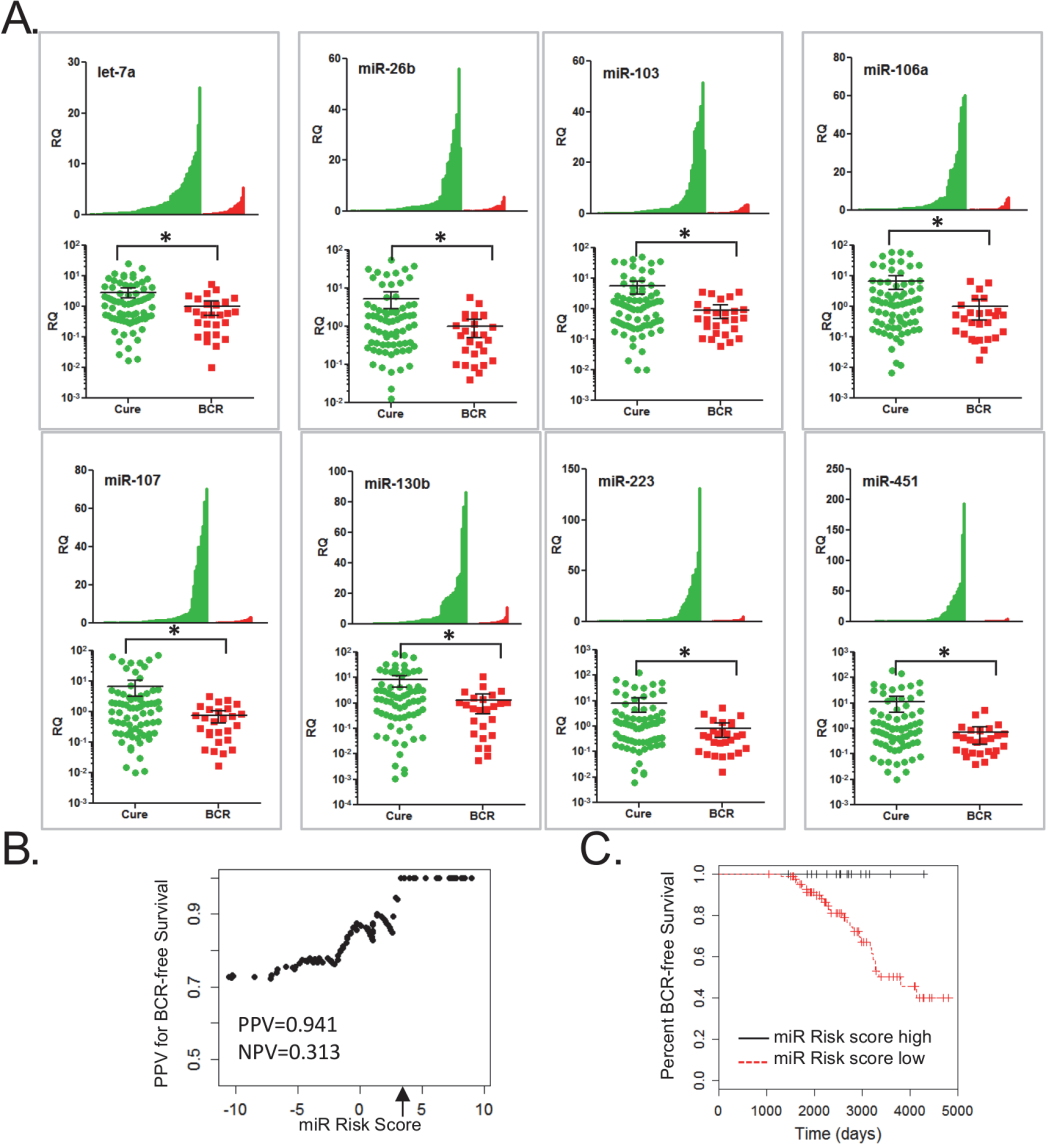
Circulating miRNAs levels in PCa patients correlate with BCR and predict disease-free survival. **A,** Waterfall (linear y-axis) and scatter (log10 y-axis) plots of let-7a, miR-26b, 103, 106a, 107, 130b, 223 and 451 levels measured by RT-qPCR in serum from all PCa patients (N = 100) grouped by cure or BCR. *p<0.05 by Wilcoxon rank sum test. **B,** NPV for disease-free survival (no BCR) in the PCa patients only (N = 100) across the range of miR Risk Scores reaching NPV = 0.941 at the miR Risk Score of 3.26 (arrow). miR Risk Score = (0.223 x miR-451) + (0.225 x miR-106a) + (0.217 x miR-223) + (0.215 x miR-107) + (0.147 x miR-130b) + (0.217 x of let-7a) + (0.196 x of miR-26b). **C,** Kaplan-Meier survival curve for all PCa patients (N = 100) above (high score) and below (low score) the miR Risk Score threshold.

Biochemical PSA recurrence (BCR) was known for the PCa patients and none of the patients received adjuvant hormone or radiotherapy prior to BCR. Although our study was not initially powered to examine BCR, the miRNA differences were so striking that also analyzed the ability of the miRNAs to predict BCR. Eight of the miRNAs were significantly lower in the PCa patients who had BCR compared to those who did not (**Fig 3A**). To test these miRNAs as a pre-surgical predictor of BCR, we calculated a miR Risk Score. NPVs were calculated across the range of risk scores to identify an optimal threshold of 3.26 that was highly predictive of disease-free survival (no BCR) (**Fig 3B**). LOOCV (NPV = 0.941, PPV = 0.313) and 2-fold CV (NPV = 1.0±0.088, PPV = 0.321±0.05) showed similar results. Across all values for the miR Risk Score, the AUC for BCR-free survival was 0.668 (**S3C Fig**).The ability of the miR Risk Score to differentiate time to BCR was examined by Kaplan-Meier curves. A “high score” or “low score” above or below the threshold predicted disease-free survival when examined in all 100 PCa patients (p = 0.031, **Fig 3C**).

### miR Scores compared to other pre-surgical clinical predictors of aggressive PCa

Logistic regression was used to determine the odds ratios (OR) for risk of harboring Gleason 4+5 PCa and BCR. Variables that are known before RP were included; initial PSA (natural logarithm), age, weight (natural logarithm), and percent positive biopsy cores. The miR Scores were included and analyzed using above/below the median (**Table 3**) as using above/below the thresholds (shown in Figs 2 and 3) resulted in an OR that approached infinity. Given that the miRs are negative predictors, the ORs are reported as low (e.g. below median) versus high (e.g. above median) for miR scores and as high versus low for the other continuous variables (i.e. PSA, weight, and age). A high miR score was a better indicator than other measured variables for absence of aggressive PCa (versus both other PCa and BPH) (**Table 3**). Similarly, a low miR score was a better indicator of BCR than other measured variables. For both aggressive PCa and BCR, the ORs based on PSA were lower but within the confidence intervals of the ORs for miR score (**Table 3**).

**Table 3:** Logistic regression analysis of pre-surgical predictors.

Predictor	Presence of Gleason 4+5 (miRScore1)		Biochemical Recurrence(miR Risk Score)	
	OR (97.5% CI)	p value	OR (97.5% CI)	p value
PSA*	5.52 (2.39, 13.46)	1.00E-04	3.21 (1.30,8.44)	0.01
Age*	2.90 (1.30,6.65)	0.01	2.71 (1.10,7.09)	0.03
Percent positive cores*	1.16 (0.42,3.15)	0.77	1.34 (0.47,4.08)	0.59
miR score**	5.77 (2.33,15.19)	2.34E-04	4.75 (1.75,14.52)	3.42E-03

*above the median

**below the median

### Prognostic miRNAs were present in prostate cells

**Fig 4:**
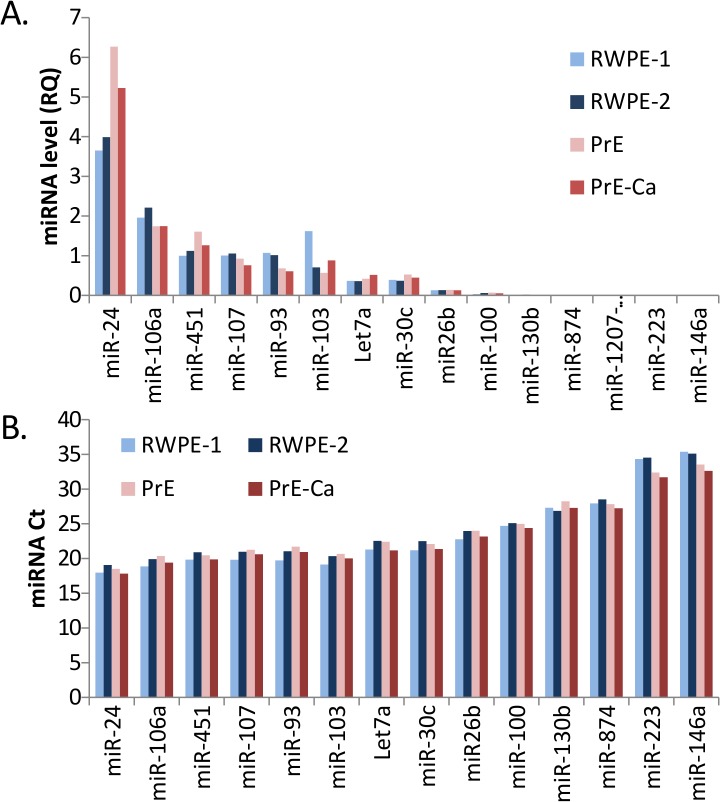
Most of the 14 miRNAs were abundant in cultured epithelial cells from both benign and malignant prostate and the expression did not vary be disease. **A,** relative quantification (RQ) of the miRNA expression in two isogenic benign/PCa pairs; RWPE-1 (benign), RWPE-2 (PCa), PrE (benign) and PrE-Ca (PCa). **B**, raw Ct values for the miRNAs in the same cells.

Biomarkers of disease may be shed from a diseased tissue to be detectable in biological fluids (e.g. PSA). To determine whether the prostate could be contributing to the miRNAs detectable in serum, the miRNA panel was analyzed in cultured prostate epithelial cells. Two different isogenic pairs of benign and PCa cells were used. RWPE-1 cells were derived from benign prostate epithelial cells and subsequently immortalized with human papilloma virus 18. RWPE-2 cells are RWPE-1 cells that were transformed with Ki-ras using the Kirsten murine sarcoma virus [30]. There was not differential expression between RWPE-1 and RWPE-2, but the miRNAs were all detectable (**Fig 4A and 4B**). Similar expression levels were seen in PrE (primary epithelial benign) and PrE-Ca (primary epithelial PCa) (**Fig 4A and 4B**). PrE and PrE-Ca cells are primary (not immortalized) were derived from areas of benign and Gleason 3 PCa from the same patient. All of the 14 miRNAs in the panel were detectable in these cells and 12 miRNAs had Cts <30; there was no difference in the miRNAs between the cell types (**Fig 4**).

## Discussion

In the present study, we obtained evidence that high serum levels of a panel of fourteen miRNAs, expressed as a combined miR Score, may have clinical utility to identify patients with low risk of harboring high-grade cancer at diagnosis. In addition, this miR Risk Score was able to identify PCa patients with a very low risk of BCR.

Our study builds on previous findings with two distinguishing elements: 1) the use of BPH as control, and 2) the thorough characterization of the BPH and PCa patients. We chose BPH as our control group rather than healthy men because BPH also raises serum PSA and is the main benign disease that triggers prostate biopsy. Furthermore, “healthy” subjects are often not age-matched to PCa patients and those that are may harbor undiagnosed BPH or PCa. Our PCa patients were selected from a cohort of >1,300 men whose prostates had been completely step-sectioned at 3-mm, permitting accurate quantitation of the Gleason grade 3, 4 and 5 components of each tumor. Because men with 100% grade 3 cancer have a very high likelihood of cure by radical prostatectomy, whereas the risk of BCR increases with each 10% increase in Gleason grade 4+5 of the tumor [7], our dichotomous cohort provided an exceptional platform for discovery and evaluation of candidate prognostic biomarkers, as the most powerful prognostic variables were already accurately known.

The combined miR Scores reflected the dramatic differences in miRNA levels and accurately identified patients without Gleason grade 4 or 5 cancer or BCR. Initial PSA and age were also significant predictors of Gleason grade 4 or 5 cancer and BCR, but the miR Scores were better predictors. Therefore, incorporation of the serum miR Scores would be useful both prior to biopsy and following a positive biopsy to identify those patients with high levels of the miRNAs who have a very low risk of harboring high-grade PCa and a low risk of BCR. This risk information is highly valuable because active surveillance or delayed treatment may well be appropriate and confidently recommended for these patients.

It is important to note that while the negative predictive ability of the miR Scores was exceedingly informative for patients with high levels of the miRNAs, but had limited use at the low end. This is reflected in the near 1 NPV and by in the strong vertical portion of our ROC curves, but then relatively poor performance in the remainder of the curves (**S3 Fig.**). For example, although men with low levels of the miRNAs would have statistically increased risk of high-grade PCa, they could actually fall into any of the disease groups.

A striking finding in our study was the uniformly low level of the 14 miRNAs in the sera from patients with high-grade cancer, whereas patients with BPH or low-grade PCa had heterogeneous and high levels of the miRNAs. This implies a systemic or prostatic reaction to high-grade PCa that reduces the amount of miRNAs in the circulation. High levels of the miRNAs were observed in a subset of both low-grade PCa and BPH cases which was an unexpected result. It is not known if circulating miRNAs are functional [31], how much the prostate contributes to serum levels of these miRNAs and if this changes in disease progression. However, we did detect many of the miRNAs in prostate-derived cells and others have detected them in benign and PCa patient tissues [32–36]. The levels of let-7a and miRs-24, -93, -106b,-130b, -146a were all lower in PCa samples compared to benign or BPH controls [32–36], although these studies did not examine the PCa by Gleason grade. This is in line with our results that the miRNAs in our panels were present at lower-levels in high-grade PCa tissues compared to benign and low-grade PCa. Twelve of the 14 miRNAs in our panel were detectable at robust levels in cultured prostate cells, both from benign and PCa tissue, suggesting that the prostate may contribute to serum levels of the miRNAs. There was not a difference in the miRNA levels in either of the isogenic benign/PCa pairs. After considering the data as a whole, we determined that since our patient data was collected from men with primary tumors, and both of these cultured PCa cells represent early PCa, one would not expect the miRNAs to be different. Thus, the cell culture findings were indeed consistent with the patient data. Considering that the serum miRNA levels were higher in men with benign or low-grade PCa and the cell culture data, if the prostate is contributing to the serum miRNAs, it does so more in benign prostate and low-grade PCa. Follow-up studies with patients after RP may reveal more about the prostatic contribution of these miRNAs to the serum.

Since commencing our study, there have been additional reports that demonstrate clinical value to circulating miRNAs in PCa. In a study similar to ours, Singh et al found that elevated levels of miRNAs-222 and -125b and decreased levels of 103 in pre-treatment serum may predict early disease recurrence [18]. Although our miR-125b findings appear to be conflict with Singh et al as we observed high-levels of miRs-125b in the patients without high-grade PCa, it is important to consider that our study was powered and designed to predict presence of high grade PCa, not BCR. Our data did emulate that of Singh et al for miR-103 as we both observed a decreased risk for BCR with low levels of miR-103. Overall, our data on BCR in this limited cohort are compelling, but a follow-up study, similar to that of Singh et al, with a larger intermediate range of Gleason scores is needed to fully assess the miR Risk Score in BCR.

We selected miR-141 based on reports that it was highly expressed in metastatic PCa [11, 13] and elevated in patients with high Gleason Scores [21]. Recent reports also show that miR-141 is higher in high-risk and patients with hormone-refractory disease [20]. However, miR-141 was undetectable in any of our patients, which may be due the age of the specimens or the fact that all the patients in our cohort had localized disease. As well, one of the miRNAs in our study, miR-451, has been consistently found in serum profiling studies and differs between healthy individuals and those with cancer [10, 37]; this miRNA was also reported to increase in hemolyzed samples [38]. In our study, none of the miRNAs, including miR-451, had differential levels in clear samples compared to those with hemolysis (**S2 Fig**).

Several of the miRNAs (miRs-106a, 93, 107, 141, 874 and 451) in our study varied in directions different from previous reports [10, 11, 13, 20]. These discrepancies may be directly attributable to biological differences in disease state and/or technical differences in detection/normalization methods. Some of these studies [11, 13, 20] examined patients with metastatic disease, while our study did not. Circulating miRNA signatures may change with the establishment of metastases, something that future studies could elaborate on. An example of detection methods differences is that our study and that of Bryant et al [11] quantified the miRNAs with Exiqon LNA technology for RT-qPCR, which does not involve a pre-amplification step, whereas microarrays and/or Taqman with pre-amplification were used in other studies [10, 13, 18, 20, 21]. Each of the methods has difference sensitivities and background.

An important factor to all profiling studies is normalization. In our study we needed to normalize miRNA levels in sera, which is not standardized and likely varies by disease. We rigorously tested serum “housekeeper” miRNAs, let7a [28] and miR-103 [27], and found them both unsuitable for our study as they varied by group (**S1B Fig**). Sanders et al also determined that let-7a was not a stable miRNA in the serum of urological cancer patients [39]. In our study RNA recovery was not altered by the year of serum collection (**S4A Fig**), but the mean miR Ct was significantly lower in the 14 samples collected prior to 1990 (**S4B Fig**). Given the small number of miRNAs measured and the striking difference between groups, we could not normalize to or adjust the results to the mean miR Ct. Moreover, none of these specific 14 samples had high levels of the miRNAs and removal of the 14 samples collected before 1990 from the analysis did not alter the results (**S4C Fig**). Therefore, we left these patients in the study.

In summary, this study used a dichotomous cohort of exceptionally well-characterized patients to identify serum miRNAs that could pre-surgically classify patients with low risk of harboring aggressive cancer or BCR. Future studies should expand these findings into a larger cohort of intermediate risk and ethnically diverse PCa patients. Our data, combined with other recently reported miRNAs [18, 21], may provide clinical benefit and improved quality of life for patients who may be spared the morbidities of PCa treatment.

## Supporting Information

S1 Dataset miRNA expression data file. Ct values from RT-qPCR.
(XLSX)

S1 Fig Normalization of serum miRNA levels. **RT-qPCR for serum miRNAs.** Relative Quantification (RQ) value determined by ddCt. A, RQ values that were used to normalize miRNA levels; total RNA input and the spike-in controls Sp3 (PCR inter-plate calibration), Sp6 (cDNA synthesis efficiency) and cel-miR-39 (RNA extraction). B, RQ values for normalizers that were not suitable for the study; let-7a, miR-103 and mean miR Ct of each sample.
(TIF)

S2 Fig Serum miRNAs and gross hemolysis of the sera. A, criteria for hemolysis. B, RT-qPCR RQ expression was log2 transformed and the median-/+ inter-quartile range for each miR is shown by hemolysis status of the serum. L = low (N = 125), I = intermediate (N = 21) and H = high (N = 16) gross hemolysis.
(TIF)

S3 Fig Receiver-operator characteristic (ROC) curves for miR Scores. ROC and AUC analysis for A, miR Score1 and low—grade PCa, B, miR Score2 and absence of high-grade PCa, C, the miR Risk Score and BCR-free survival.
(TIF)

S4 Fig Removal of older serum sample does not alter results. Analysis by year of serum collection between the groups by, A, RNA recovery, and, B, mean miR Ct (log2). C, analysis of miRNAs by group after removal of 14 patients with serum collections ≤1990; RQ on y-axis. *p<0.05, ** p<0.01, *** p<0.0001 by Kruskal–Wallis one-way analysis of variance with Dunn's multiple comparisons test.
(TIF)
