# Supplementary figures and images for: Elevated Serum MicroRNA Levels Associate with Absence of High-Grade Prostate Cancer in a Retrospective Cohort
Source: PLoS One. 2015 Apr 14;10(4):e0124245. doi: 10.1371/journal.pone.0124245 (PMC4396984; doi:10.1371/journal.pone.0124245)

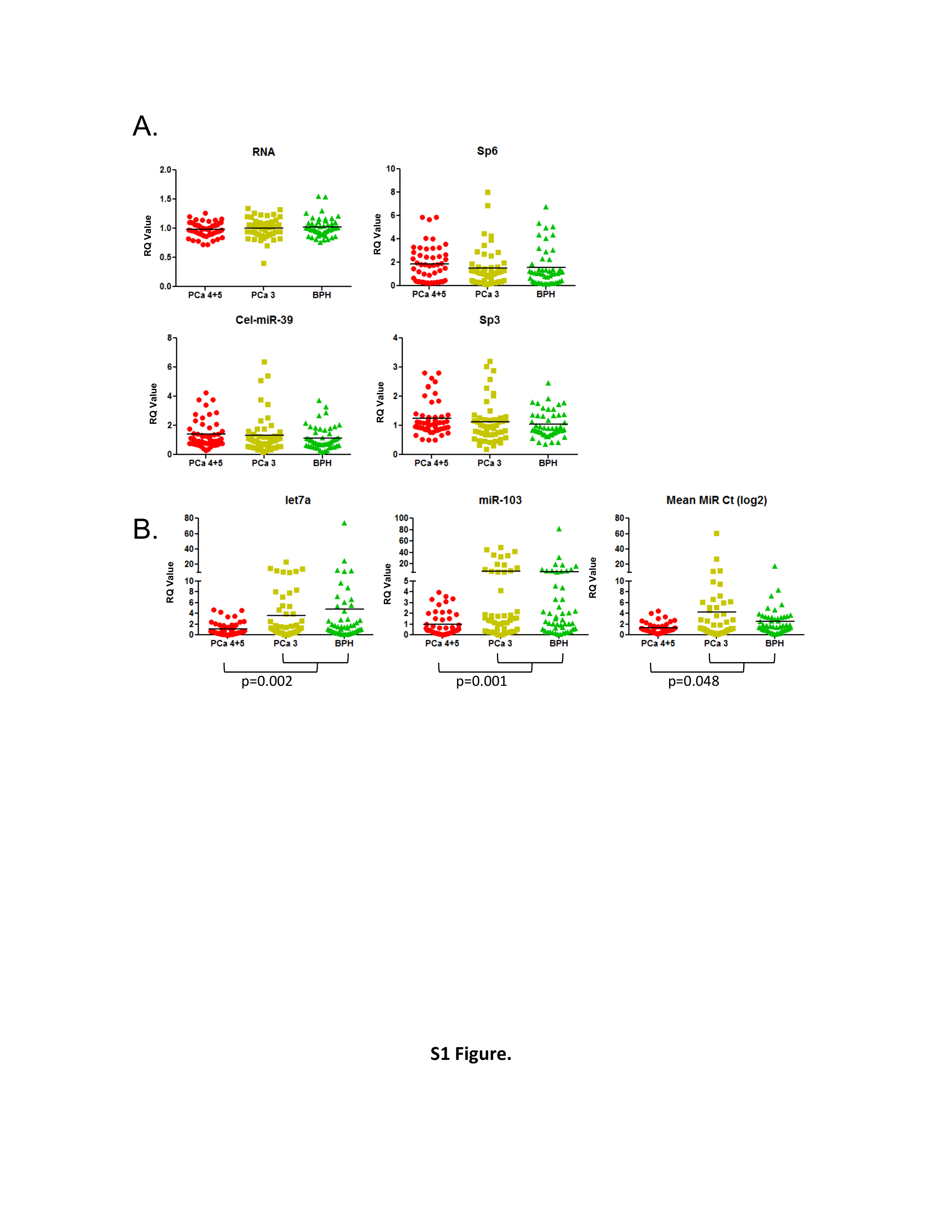

Supplement: S1 Fig — RT-qPCR for serum miRNAs. Relative Quantification (RQ) value determined by ddCt. A, RQ values that were used to normalize miRNA levels; total RNA input and the spike-in controls Sp3 (PCR inter-plate calibration), Sp6 (cDNA synthesis efficiency) and cel-miR-39 (RNA extraction). B, RQ values for normalizers that were not suitable for the study; let-7a, miR-103 and mean miR Ct of each sample. (TIF) [file pone.0124245.s002.tif]

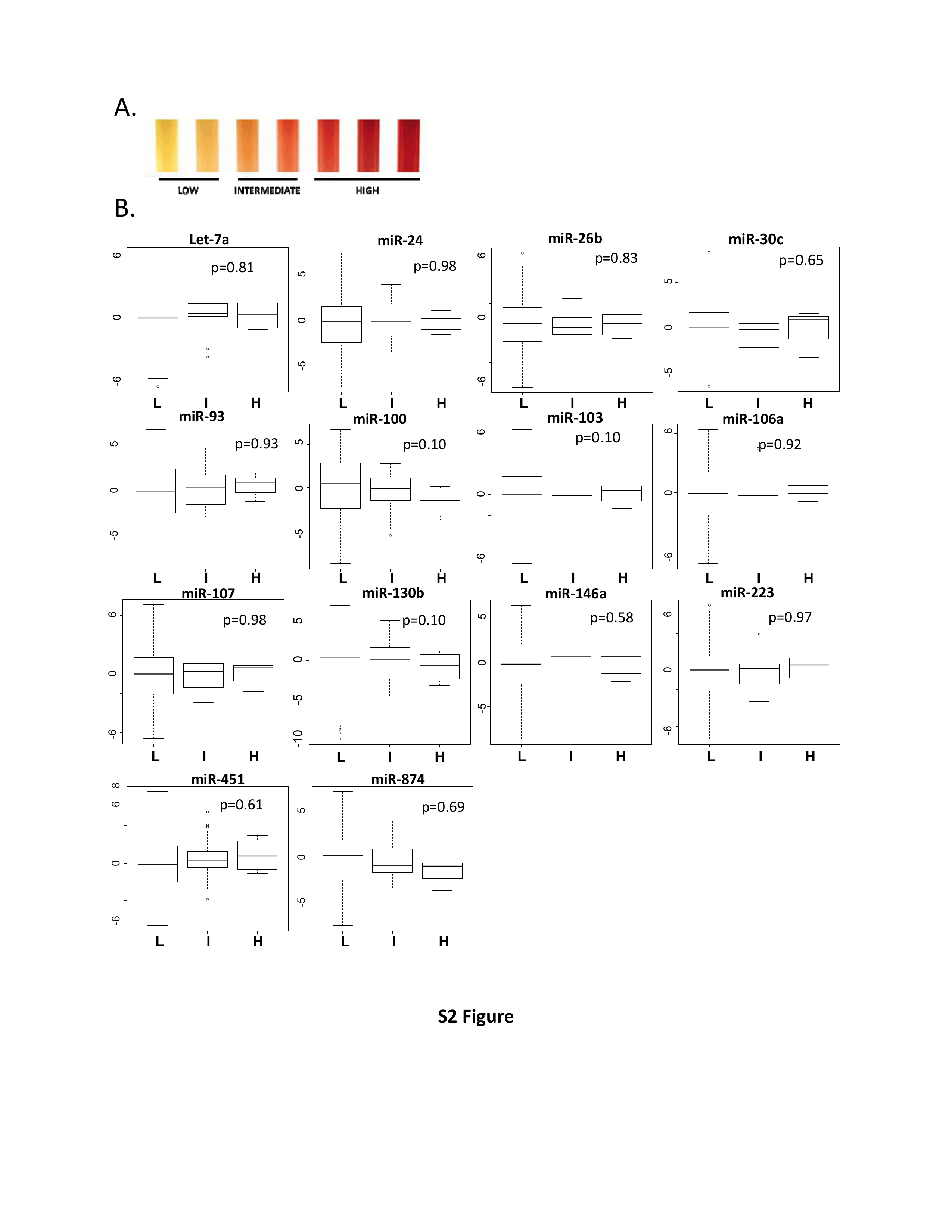

Supplement: S2 Fig — A, criteria for hemolysis. B, RT-qPCR RQ expression was log2 transformed and the median-/+ inter-quartile range for each miR is shown by hemolysis status of the serum. L = low (N = 125), I = intermediate (N = 21) and H = high (N = 16) gross hemolysis. (TIF) [file pone.0124245.s003.tif]

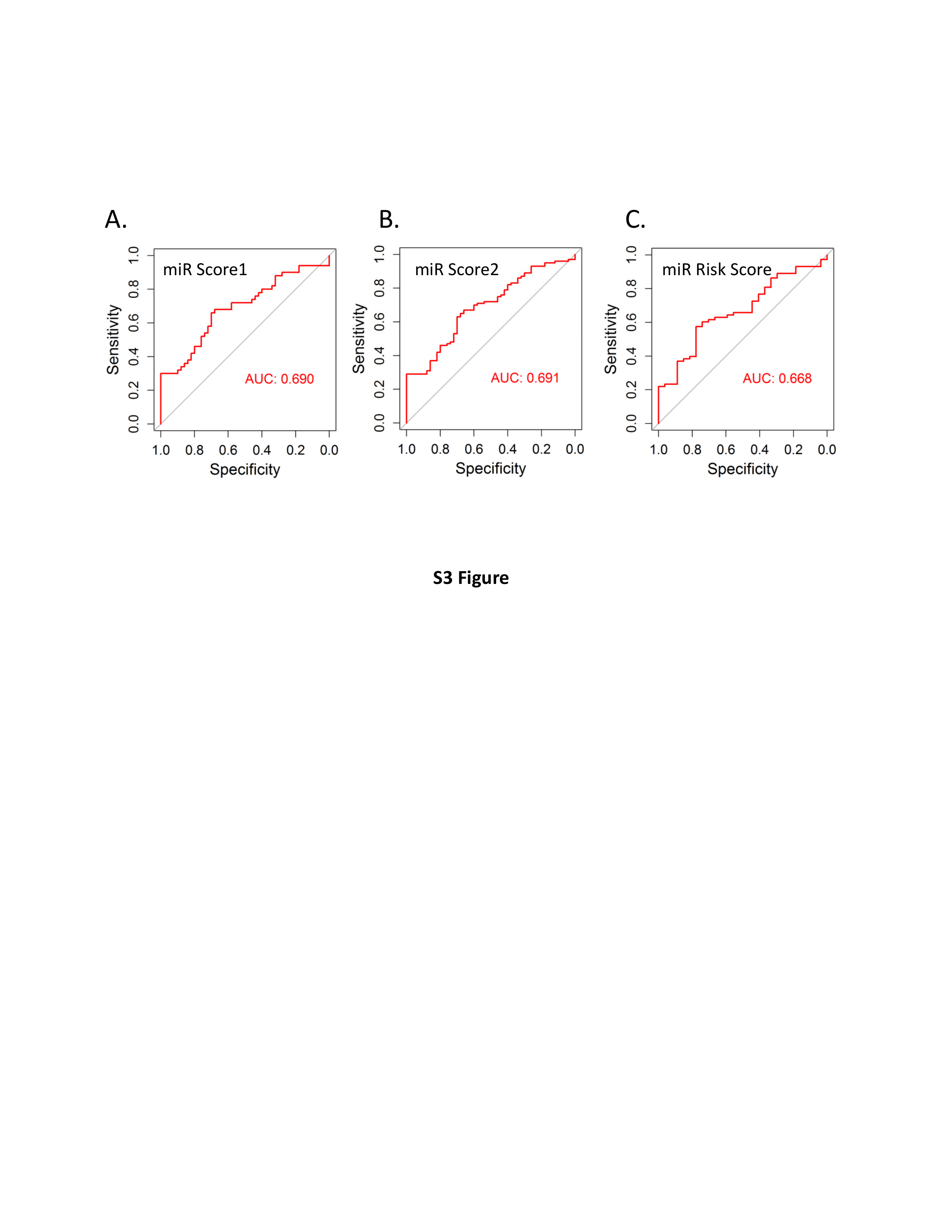

Supplement: S3 Fig — ROC and AUC analysis for A, miR Score1 and low—grade PCa, B, miR Score2 and absence of high-grade PCa, C, the miR Risk Score and BCR-free survival. (TIF) [file pone.0124245.s004.tif]

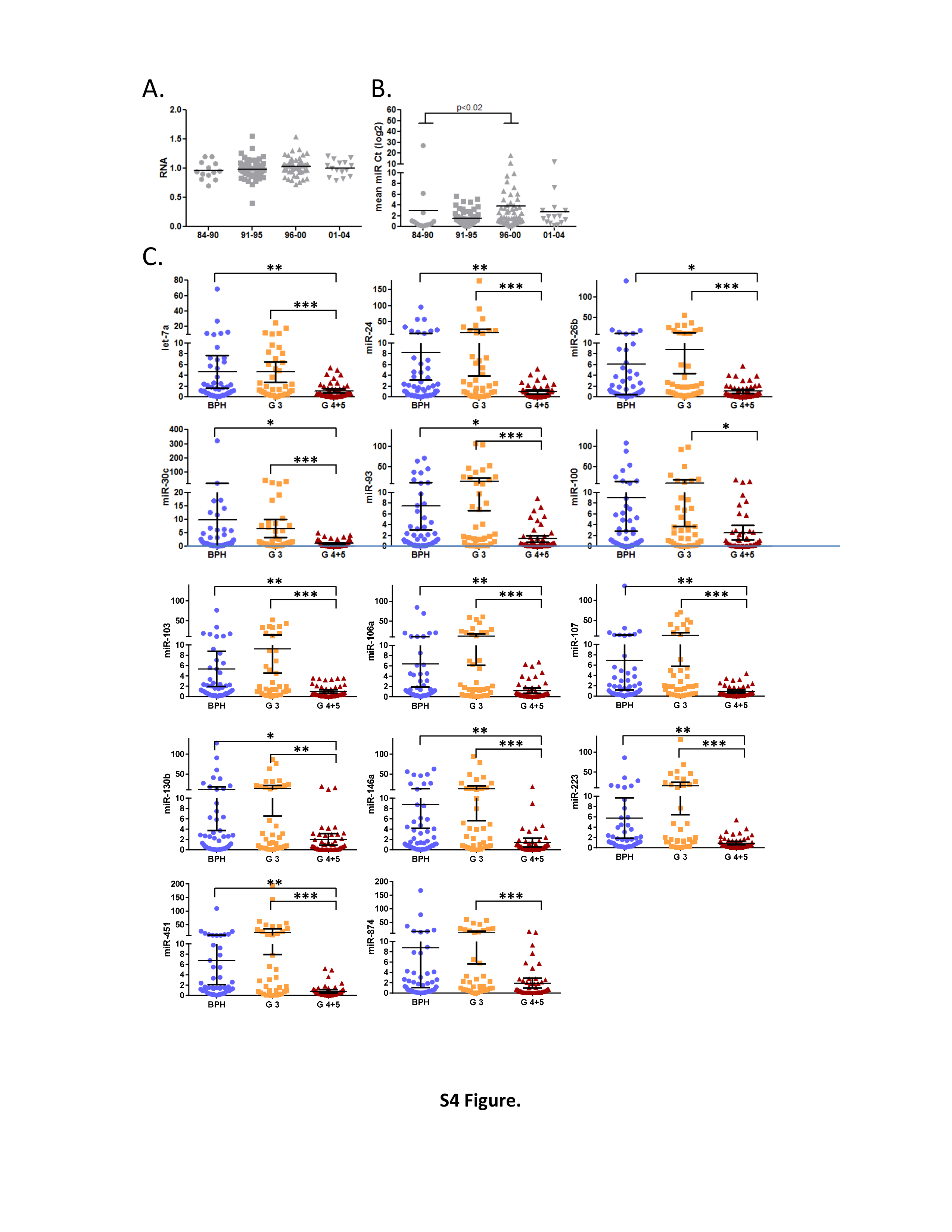

Supplement: S4 Fig — Analysis by year of serum collection between the groups by, A, RNA recovery, and, B, mean miR Ct (log2). C, analysis of miRNAs by group after removal of 14 patients with serum collections ≤1990; RQ on y-axis. *p<0.05, ** p<0.01, *** p<0.0001 by Kruskal–Wallis one-way analysis of variance with Dunn's multiple comparisons test. (TIF) [file pone.0124245.s005.tif]
